# Dabie bandavirus identified from patients with severe fever with thrombocytopenia syndrome in northwestern of Hubei Province, China, 2024

**DOI:** 10.3389/fpubh.2025.1666857

**Published:** 2025-10-29

**Authors:** Mengzhu Zhang, Peixi Fu, ZhongJi Meng, Yuqian He, Xueqin Qin, Sen Luo, Yunzhen Xu, Li Liu, Guangyu Qiu, Yang Liu, Yanli Peng, Fangmin Song, Tianyi Xu, Jiao Yin, Mingming Liu, Chuanmin Wang

**Affiliations:** ^1^Department of Infectious Diseases, Institute of Biomedical Research, Regulatory Mechanism and Targeted Therapy for Liver Cancer Shiyan Key Laboratory, Hubei Provincial Clinical Research Center for Precise Diagnosis and Treatment of Liver Cancer, Taihe Hospital, Hubei University of Medicine, Shiyan, China; ^2^Department of Emergency, Xiangyang Central Hospital, Afffliated Hospital of Hubei University of Arts and Science, Xiangyang, China; ^3^Department of Infectious Diseases, Yunyang People’s Hospital, Shiyan, China; ^4^Department of Infectious Diseases, Yunxi People’s Hospital, Shiyan, China; ^5^School of Basic Medicine, Hubei University of Arts and Science, Xiangyang, China

**Keywords:** SFTS, DBV, clinical parameters, genotypes, Hubei Province

## Abstract

**Introduction:**

Severe fever with thrombocytopenia syndrome (SFTS), caused by Dabie bandavirus (DBV), is a zoonotic disease characterized by substantial mortality. Hubei Province is an epidemic region with a high incidence rate of SFTS. The clinical manifestations and case fatality rates (CFRs) of SFTS correlate with specific geographic regions and genotypes of DBV.

**Methods:**

From January to December 2024, serum samples were obtained from 69 patients with suspected DBV infection in northwestern Hubei Province, China. The presence of DBV RNA was used as the diagnostic criterion for SFTS. Demographic characteristics, clinical manifestations, and laboratory findings of confirmed SFTS patients were systematically collected. Phylogenetic analyses of the DBV L, M, and S gene segments were performed using the maximum likelihood method to elucidate the genetic diversity of the viral isolates.

**Results:**

A total of 19 patients with confirmed SFTS were identified in northwestern Hubei Province in 2024, with a CFR of 31.6% (6/19). Clinical analyses indicated that bleeding, disturbance of consciousness, prolonged activated partial thromboplastin time (APTT), elevated blood urea nitrogen (BUN), and high viral load (≥10^7^ copies/mL) were critical prognostic indicators of disease severity. Maximum likelihood phylogenetic analyses of the L, M, and S gene segments demonstrated that genotype F was the predominant lineage circulating in this region.

**Discussion:**

This study delineates the genomic diversity and genotype distribution of circulating DBV strains, providing insights into viral etiology in northwestern Hubei. Furthermore, specific clinical/laboratory markers may signal adverse outcomes, emphasizing the imperative for symptom recognition and dynamic monitoring of critical laboratory parameters.

## 1 Introduction

Severe fever with thrombocytopenia syndrome (SFTS) is an emerging viral infectious disease caused by Dabie bandavirus (DBV), previously known as severe fever with thrombocytopenia syndrome virus (SFTSV) (1). The pathogen was first isolated from the sera of febrile patients exhibiting leukopenia and thrombocytopenia in central China in 2009 (2). The primary mode of transmission for DBV is through tick bites, with *Haemaphysalis longicornis* being the most likely vector (3). DBV targets lymph nodes near the site of tick bite entry, impairs adaptive immunity by suppressing B-cell antibody production, promotes viral spread, and triggers a systemic cytokine storm due to dysregulated immune signaling (4).

SFTS is characterized by persistent high fever, thrombocytopenia, leukopenia, gastrointestinal symptoms, and liver dysfunction (5). In severe cases, it can progress to multiple organ dysfunction, including myocarditis, acute kidney failure, pulmonary injury, and central nervous system involvement (6). Studies indicate that the case–fatality ratio (CFR) of SFTS ranges from 5.11 to 44.7% (7). The disease remains endemic in East Asia, predominantly affecting central and eastern China, rural South Korea, and western Japan. Its geographic distribution has expanded to other Asian regions, including Myanmar, Thailand, Vietnam, and Pakistan, suggesting a progressive spatial dissemination of this arboviral disease (8–11). Given its high CFR and widespread distribution of transmission vectors, SFTS has become a significant public health concern.

DBV, a member of the Phlebovirus genus within the Bunyaviridae family, is a spherical, enveloped virus with a negative-sense single-stranded RNA genome. It has a diameter of approximately 80–120 nm. The genome consists of three RNA segments: the large (L) segment encodes the RNA-dependent RNA polymerase (RdRp), the medium (M) segment encodes the Gn and Gc glycoproteins, and the small (S) segment encodes both the nucleoprotein (NP) and the nonstructural protein (NS) (12). Each of the three viral genomic segments can be independently classified into distinct phylogenetic lineages. Currently, there is no universal standard or nomenclature for DBV genotyping (12). Takahashi et al. established the C-J classification system, categorizing strains into Chinese and Japanese clusters on the basis of geographic distribution, whereas Liu et al. proposed a genotypic classification into five genotypes (A–E) (13, 14). The most widely adopted genotyping method remains the A-F classification system introduced by Fu et al. (12, 15). The distribution of DBV genotypes demonstrates significant geographic clustering heterogeneity, and the regional prevalence of specific genotypes may be a determinant contributing to cross-national disparities in case fatality rates CFR. Genotypes F (43.6%), A (20.1%), and D (15.4%) cocirculate across broad geographic regions in mainland China, resulting in relatively lower CFRs (15, 16). In contrast, genotype B, which is predominant in South Korea and Japan, is associated with the highest prevalence and CFR (17). Recent surveillance has detected genotype B in China’s Zhoushan Archipelago and Taiwan region, highlighting its expanding geographic range (18).

Northwestern Hubei Province, with its mountainous or hilly terrain, dense vegetation, and hot, humid summers, offers ecological and biological conditions that are conducive to tick proliferation. Agricultural workers and outdoor laborers in Hubei, a major agricultural province with a large rural population primarily engaged in farming and animal husbandry, face increased risks of DBV infection (19). This elevates the regional susceptibility to infections. Nevertheless, the limited health care resources in rural areas can result in delayed reporting and diagnosis of cases, which may undermine the accuracy of epidemiological surveillance data.

This study collected serum samples from suspected SFTS patients in northwestern Hubei Province, analyzed the clinical characteristics of SFTS patients and the phylogenetic features of DBV in this region, and further investigated the associations between DBV genotypes and disease severity.

## 2 Materials and methods

### 2.1 Ethics statement

The samples analyzed were obtained prior to the study beginning with epidemiological surveillance activities conducted for SFTS case management. All the biological materials were processed in an anonymized manner to protect participant confidentiality. The investigation protocol was granted ethical approval by the Institutional Review Board at Taihe Hospital of Hubei University of Medicine (Approval Code: 2025KS071), with strict compliance with China’s Guidelines for Ethical Review of Biomedical Research Involving Human Subjects.

### 2.2 Clinical sample collection

This study enrolled patients clinically diagnosed with suspected SFTS at the Taihe Hospital of Hubei University of Medicine from January to December 2024. Participants meeting the SFTS diagnostic criteria established by the Chinese National Health Commission were included as confirmed cases. Specifically, viral RNA was extracted from serum specimens of suspected SFTS patients using RNA extraction kits, with DBV RNA positivity serving as the diagnostic criterion. Telephone follow-up assessments were conducted within 1 month postdischarge for all discharged patients. All laboratory parameters and serum samples were collected during the acute disease phase.

Whole blood samples were collected into microcentrifuge tubes without anticoagulant and allowed to clot at room temperature for 3 h. Following complete coagulation, the samples were centrifuged at 3000 × g for 10 min at 4 °C. The serum was then carefully aspirated using sterile transfer pipettes, immediately aliquoted, and stored at −80 °C until subsequent analysis.

### 2.3 Clinical data collection

The demographic characteristics, clinical manifestations, and laboratory findings of confirmed SFTS patients in the northwestern region of Hubei Province were collected by accessing the electronic medical record (EMR) system of the Taihe Hospital of Hubei University of Medicine, with data specifically extracted from the initial 24-h admission period.

### 2.4 RNA extraction and DBV detection

Viral RNA was extracted from the serum samples of suspected SFTS patients using a nucleic acid extraction kit (DA3040, Guangzhou DAAN Gene Co., Ltd., China). The quantitative detection of DBV was subsequently performed using the Severe Fever with Thrombocytopenia Syndrome Bunyavirus Nucleic Acid Detection Kit (PCR-fluorescent probe method) with PCR Reagent (DA0340, Guangzhou DAAN Gene Co., Ltd., China) in conjunction with the Cobas z 480 fully automated fluorescence PCR analyzer (Roche, Switzerland). This kit employs one-step real-time fluorescent PCR technology with specific primers and fluorescent probes designed to target highly conserved regions within the coding region of the DBV gene. Quantitative detection of DBV RNA in serum samples was achieved by one-step RT–PCR amplification, and the viral load was calculated using the following formula: 10^x^TCID_50_/mL = 0.496 × 10^x + 3^copies/mL.

### 2.5 DBV whole-genome sequencing and analysis

DBV RNA-positive samples were sequenced by Sangon Biotech (Shanghai) Co., Ltd. The next generation sequencing (NGS) data were assembled using SPAdes, followed by gap filling in the assembled alleles with Gap Filler. A comprehensive phylogenetic analysis of DBV strains was conducted using MEGA X software to determine viral genotypes and elucidate their evolutionary relationships. The analysis was performed using maximum likelihood (ML) based on the aligned nucleotide sequences of the L, M, and S segments, with robustness assessed by 1,000 bootstrap replicates. The analysis encompassed the coding nucleotide sequences of the L, M, and S genomic segments from three DBV isolates identified in this study, along with 38 reference DBV sequences retrieved from the GenBank database.

## 3 Results

### 3.1 Basic information of the SFTS patients

From January to December 2024, 69 suspected SFTS patients clinically diagnosed at Taihe Hospital of Hubei University of Medicine were investigated. Real-time PCR confirmed DBV infection in 19 patients (7 males and 12 females) of whom 16 were from Shiyan city and three from Xiangyang city in the Hubei Province. The outcomes included 13 survivors and 6 fatalities, with ages ranging from 52 to 79 years (Table 1).

**Table 1:** Demographic information, clinical manifestations, and laboratory findings on the first day of admission for patients diagnosed with SFTS in Northwest Hubei.

Case number	Age (years)/sex	District	Outcome	Clinical manifestations					Laboratory findings					
				Maximum temperature (37.5–42 °C)	Respiratory symptoms	Digestive symptoms	Bleeding	Conscious disturbance	WBC (×10^9^/L)	PLT (×10^9^/L)	AST (U/L)	APTT (s)	BUN (mmol/L)	Viral load (copies/ml)
1	77/F	Shiyan	Survival	38.0	Yes	Yes	No	No	1.94	94	86.7	33.1	4.78	1.35 × 10^5^
2	73/F	Shiyan	Survival	40.0	No	Yes	No	No	3.65	65	132.0	33.1	3.96	1.00 × 10^3^
3	59/F	Shiyan	Survival	39.0	No	Yes	Yes	No	1.56	31	483.8	51.1	2.95	7.04 × 10^5^
4	72/F	Shiyan	Death	39.0	Yes	Yes	Yes	Yes	3.25	97	497.7	62.3	5.29	1.39 × 10^9^
5	52/F	Shiyan	Survival	40.0	Yes	Yes	No	No	0.84	50	343.3	39.2	2.72	4.83 × 10^5^
6	57/F	Xiangyang	Survival	42.0	Yes	Yes	Yes	No	5.57	25	59.4	41.8	8.15	1.28 × 10^3^
7	79/F	Shiyan	Death	37.5	Yes	No	Yes	Yes	2.38	47	499.0	55.4	9.52	2.08 × 10^5^
8	56/M	Shiyan	Survival	39.5	No	Yes	No	No	5.36	34	371.7	50.4	3.25	3.62 × 10^6^
9	56/F	Shiyan	Survival	39.2	No	Yes	No	No	0.86	28	317.1	37.6	4.77	1.26 × 10^6^
10	58/F	Shiyan	Survival	39.0	No	Yes	No	No	0.64	30	202.1	42.2	3.7	3.33 × 10^6^
11	62/M	Shiyan	Survival	39.0	Yes	Yes	Yes	No	2.69	14	471.7	45.8	4.75	3.93 × 10^5^
12	72/M	Xiangyang	Death	39.5	No	Yes	Yes	Yes	1.30	33	510.0	50.6	17.3	2.91 × 10^10^
13	63/F	Shiyan	Survival	38.8	Yes	Yes	No	No	2.32	38	367.3	35.6	3.98	6.99 × 10^4^
14	79/M	Shiyan	Death	38.8	No	Yes	Yes	Yes	1.37	21	227.1	38.7	10.0	2.21 × 10^9^
15	58/F	Shiyan	Survival	39.3	Yes	Yes	No	No	1.97	86	44.7	35.2	4.48	5.01 × 10^3^
16	72/F	Xiangyang	Survival	38.0	No	Yes	Yes	No	2.88	51	282.0	51.3	4.69	1.40 × 10^3^
17	73/M	Shiyan	Death	38.8	Yes	Yes	Yes	Yes	1.80	79	117.0	46.1	6.9	1.10 × 10^8^
18	60/M	Shiyan	Death	38.3	No	Yes	Yes	Yes	3.06	36	600.9	67.5	7.93	1.40 × 10^9^
19	63/M	Shiyan	Survival	38.5	No	Yes	Yes	No	2.03	43	645.0	59.8	9.33	3.18 × 10^5^

Maximum temperature, Armpit temperature; Respiratory symptoms, Cough, sputum or dyspnea; Digestive symptoms, Nausea, vomiting or diarrhea; WBC, white blood cell; PLT, platelet; AST, aspartate aminotransferase; APTT, activated partial thromboplastin time; BUN, blood urea nitrogen.

### 3.2 Clinical characteristics of SFTS patients

The clinical presentations of SFTS patients included fever, respiratory symptoms, digestive symptoms, bleeding, and disturbance of consciousness. Among the 19 confirmed SFTS patients, the predominant presentations were fever (100%, 19/19), digestive symptoms (94.7%, 18/19), bleeding (57.9%, 11/19), respiratory symptoms (47.4%, 9/19), and disturbance of consciousness (31.6%, 6/19) (Table 2). In the death group (n = 6), all patients presented with fever (100%, 6/6), bleeding (100%, 6/6), and disturbance of consciousness (100%, 6/6), whereas half presented with digestive symptoms (50.0%, 3/6) (Table 2). Among the survivor group (n = 13), the clinical presentations ranked by frequency of occurrence were fever (100%, 13/13), digestive symptoms (100%, 13/13), respiratory symptoms (46.2%, 6/13), and bleeding (38.5%, 5/13), with no disturbance of consciousness (Table 2). The case–control analysis revealed that patients in the death group had significantly higher incidence rates of bleeding and disturbance of consciousness than did those in the survivor group (*p* < 0.05).

**Table 2:** Characteristics of clinical manifestations and laboratory findings on admission of SFTS patients with different outcomes (death or survival).

Parameter	Total (19 cases)	Death group (6 cases)	Survivor group (13 cases)	*p*-value
Clinical manifestations				
Fever	100% (19/19)	100% (6/6)	100% (13/13)	1.000
Respiratory symptoms	47.4% (9/19)	50.0% (3/6)	46.2% (6/13)	0.876
Digestive symptoms	94.7% (18/19)	83.3% (5/6)	100% (13/13)	0.130
Bleeding	57.9% (11/19)	100% (6/6)	38.5% (5/13)	**0.012**
Disturbance of consciousness	31.6% (6/19)	100% (6/6)	0% (0/13)	**<0.001**
Laboratory findings				
WBC (×10^9^/L)	2.39 ± 1.37	2.19 ± 0.84	2.49 ± 1.57	0.678
PLT (×10^9^/L)	47.47 ± 25.10	52.17 ± 29.51	45.31 ± 23.78	0.594
AST(U/L)	329.40 ± 188.05	408.62 ± 190.44	292.83 ± 182.61	0.222
APTT(s)	46.15 ± 10.17	53.43 ± 10.58	42.79 ± 8.35	**0.029**
BUN (mmol/L)	6.23 ± 3.54	9.49 ± 4.20	4.73 ± 1.93	**0.003**
Viral load (>10^7^copies/mL)	26.3% (5/19)	83.3% (5/6)	0% (0/13)	**0.000**

The bold values in the table denote statistically significant clinical parameters (*p* < 0.05).

### 3.3 Laboratory findings for SFTS patients

Laboratory indicators such as white blood cell (WBC) count, platelet (PLT) count, aspartate aminotransferase (AST) level, activated partial thromboplastin time (APTT), blood urea nitrogen (BUN), and viral load were measured in 19 patients with SFTSs on the first day of hospitalization (Table 1). The primary laboratory findings revealed thrombocytopenia (100%, 19/19) and elevated AST levels (100%, 19/19) (Table 1). Notably, the viral load ranged from 1.00 × 10^3^ to 2.91 × 10^10^ copies per milliliter of serum. No statistically significant differences were detected in white blood cell counts (2.19 ± 0.84 × 10^9^/L vs. 2.49 ± 1.57 × 10^9^/L, *p* = 0.678), platelet counts (52.17 ± 29.51 × 10^9^/L vs. 45.31 ± 23.78 × 109/L, *p* = 0.594), or AST levels (408.62 ± 190.44 U/L vs. 292.83 ± 182.61 U/L, *p* = 0.222) between the death and survivor groups (Table 2). However, compared with the survivor group, the death group presented a significantly prolonged APTT (53.43 ± 10.58 s vs. 42.79 ± 8.35 s, *p* = 0.029). Additionally, the serum BUN level was significantly greater in the patients who died than in those who survived (9.49 ± 4.20 mmol/L vs. 4.73 ± 1.93 mmol/L, *p* = 0.003) (Table 2). Notably, five of the six nonsurvivors presented viral loads exceeding 10^7^ copies/mL, whereas all survivors maintained viral loads below this critical threshold (83.3% vs. 0%, *p* = 0.000) (Table 2).

### 3.4 Sequences of the isolated DBV isolates

Three full-length DBV genomes were obtained from 19 confirmed SFTS patients: HBSY2024-4, HBSY2024-14, and HBSY2024-18. The nucleotide homology of the L, M, and S segments of the three isolates was greater than 99.66% compared with the sequences available in GenBank using the Basic Local Alignment Search Tool (BLAST) (Table 3). The amino acid sequences of the L, M, S (NS), and S (NP) segments across the three DBV strains presented greater than 99.81% identity when analyzed using BLAST.

**Table 3:** Sequence information of isolated DBV strains.

Case number	Strain	GenBank accession no. (L/M/S)	Genotype (L/M/S)	Sequence identity (%)							
				L		M		S (NS)		S (NP)	
				Nucleotide	AA	Nucleotide	AA	Nucleotide	AA	Nucleotide	AA
4	HBSY2024-4	PQ865996/PQ865999/PQ866002	F/F/F	99.66	100	99.78	99.81	99.66	99.66	100	100
14	HBSY2024-14	PQ865997/PQ866000/PQ866003	F/F/F	99.73	99.86	99.72	99.91	100	100	99.86	100
18	HBSY2024-18	PQ865998/PQ866001/PQ866004	F/F/F	99.87	100	99.72	99.91	99.66	100	99.86	100

NS, nonstructural protein; NP, nucleocapsid protein; AA, amino acid.

### 3.5 Phylogenetic analysis of DBV

**Figure 1:**
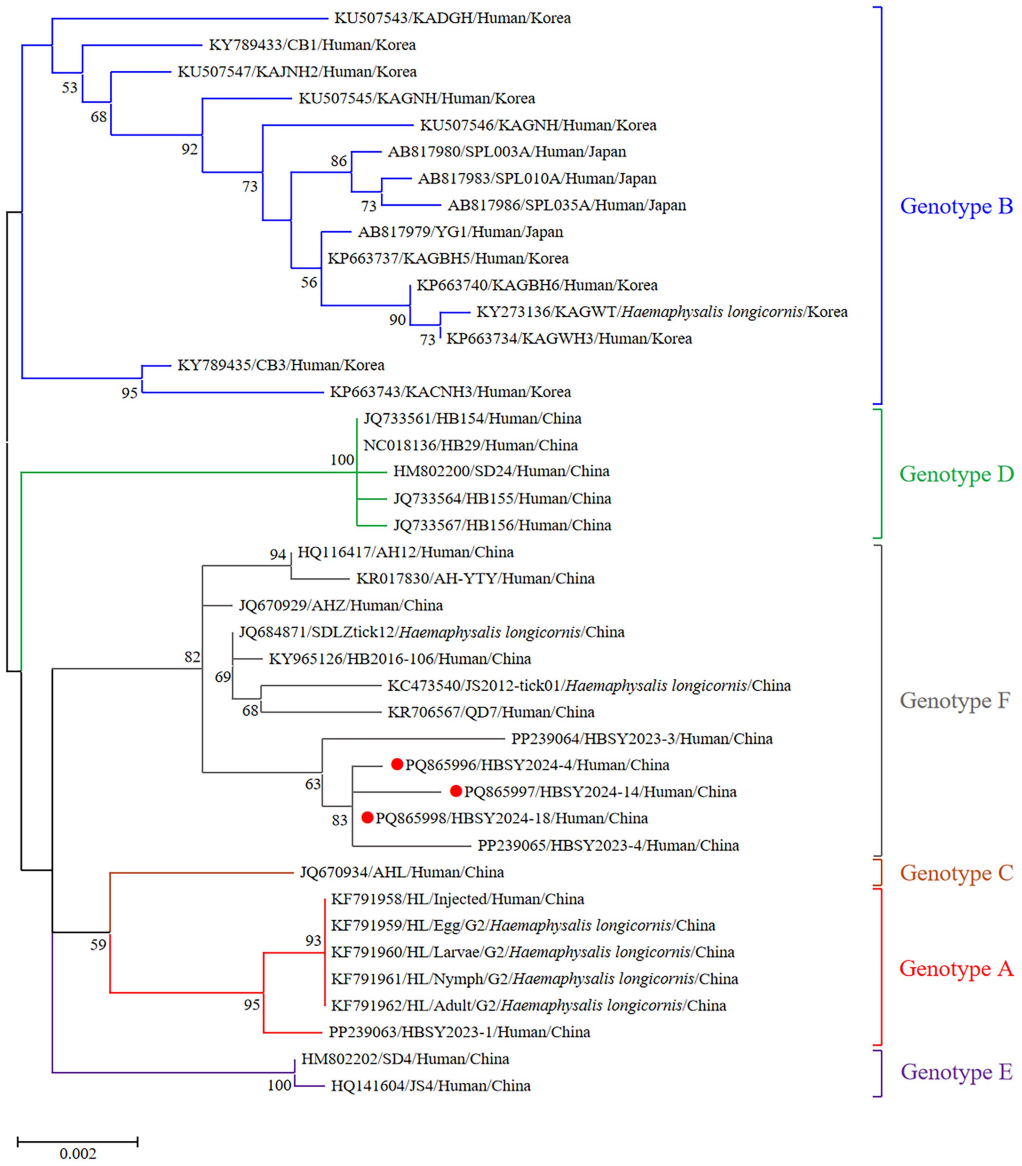
Phylogenetic analysis based on the complete ORF sequences of the L segment of the DBV isolates using the maximum likelihood (ML) method and the Tamura-Nei model. The phylogenetic branches were supported with bootstrap values greater than 50%. The numbers on the branches indicate bootstrap percentages based on 1,000 replications. The red, blue, brown, green, purple, and gray branches were designated as DBV isolates belonging to genotypes A, B, C, D, E, and F, respectively. The DBV sequences determined in this study are shown as red closed circles.

**Figure 2:**
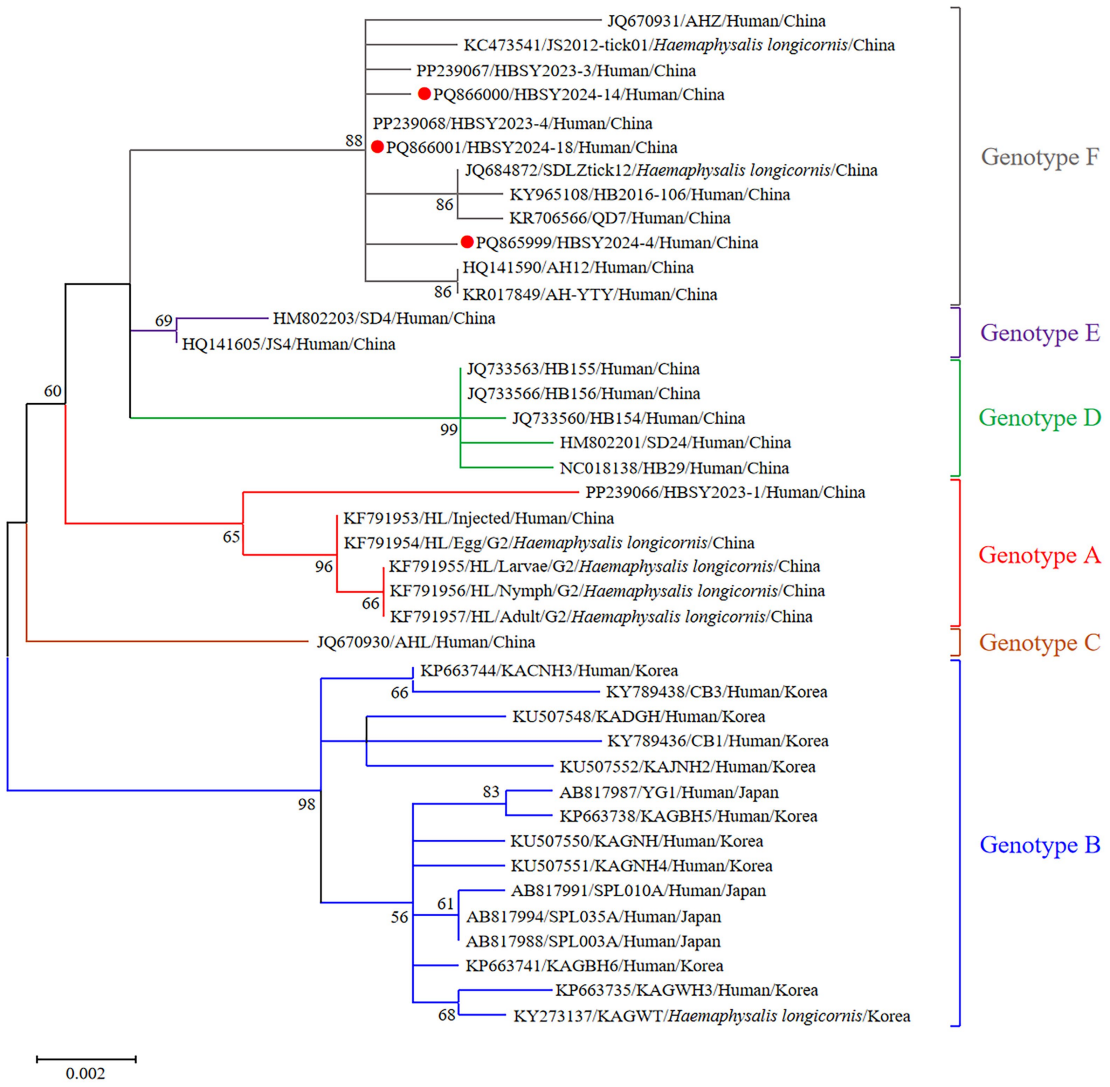
Phylogenetic analysis based on the complete ORF sequences of the M segment of DBV isolates using the maximum likelihood (ML) method and the Hasegawa-Kishino-Yano model. The phylogenetic branches were supported with bootstrap values greater than 50%. The numbers on the branches indicate bootstrap percentages based on 1,000 replications. The red, blue, brown, green, purple, and gray branches were designated as DBV isolates belonging to genotypes A, B, C, D, E, and F, respectively. The DBV sequences determined in this study are shown as red closed circles.

**Figure 3:**
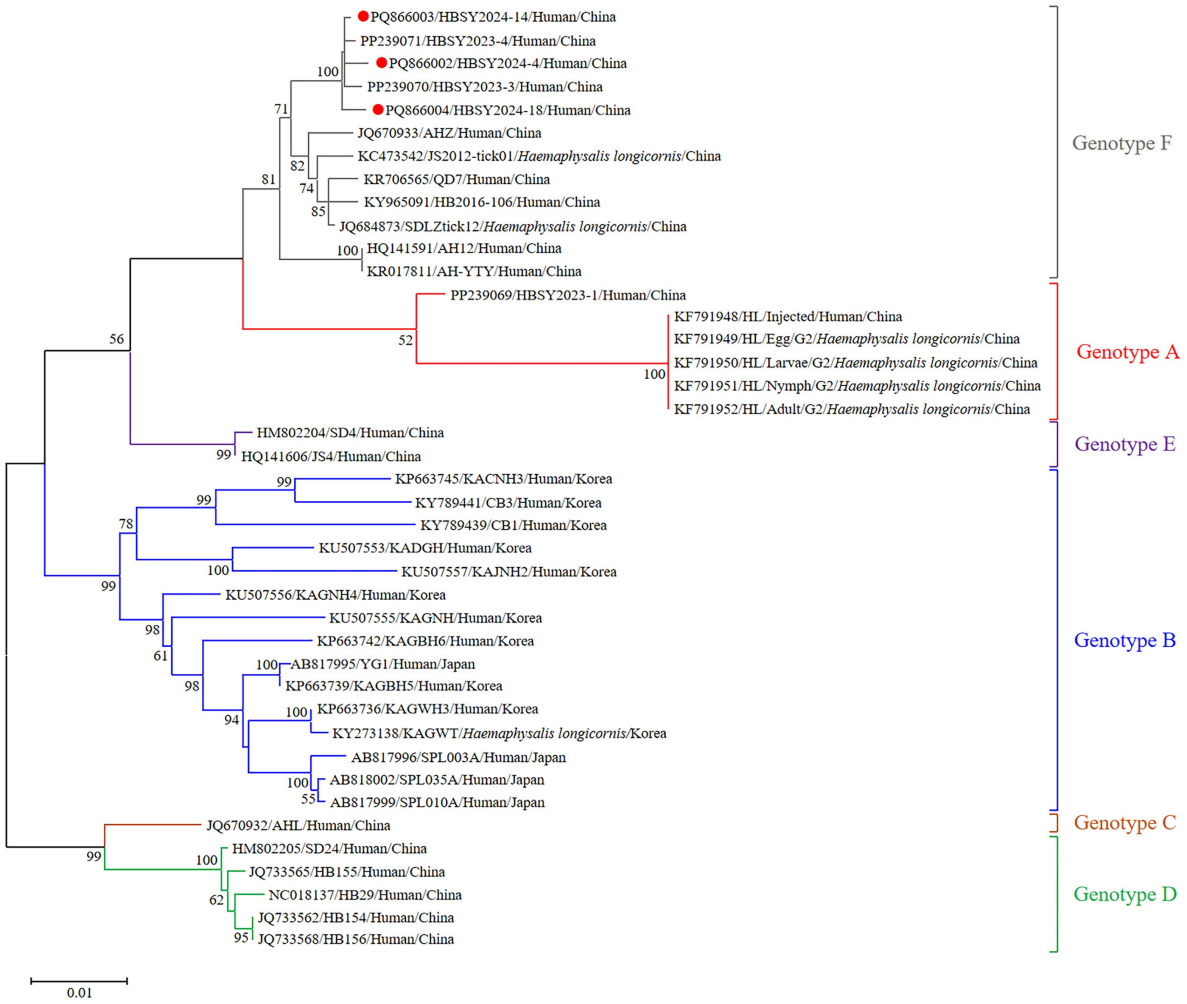
Phylogenetic analysis based on the complete ORF sequences of the S segment of the DBV isolates via the maximum likelihood (ML) method based on the Kimura 2-parameter model. The phylogenetic branches were supported with bootstrap values greater than 50%. The numbers on the branches indicate bootstrap percentages based on 1,000 replications. The red, blue, brown, green, purple, and gray branches were designated as DBV isolates belonging to genotypes A, B, C, D, E, and F, respectively. The DBV sequences determined in this study are shown as red closed circles.

The DBV sequences of the L, M, and S fragments of HBSY2024-4, HBSY2024-14, and HBSY2024-18 from Shiyan city were uploaded to the GenBank database with accession numbers PQ865996-PQ866004 (Table 3). The L, M, and S fragments of the DBV isolates from these three strains all clustered within the genotype F clade (Figures 1–3).

## 4 Discussion

Our study revealed that patients with STFS who display hemorrhagic symptoms and neurological involvement have a significantly higher mortality rate than do those without these symptoms. Bleeding manifestations primarily included gingival bleeding and subcutaneous ecchymosis, with one patient exhibiting fecal occult blood positivity. Notably, all six patients who developed consciousness impairment also experienced delirium, likely due to cytokine storm-induced vascular hyperpermeability, which facilitates the neuroinvasion of DBV across the blood–cerebrospinal fluid barrier, resulting in viral encephalitis. Previous evidence suggests that patients with SFTS who develop concurrent multiorgan failure and neurological symptoms have fatality rates of up to 44.7% (20). A murine model study investigating the neuropathological mechanisms of DBV-induced encephalopathy demonstrated dynamic viral kinetics postneurotropic infection, with viral loads exhibiting exponential increases at 24 h postinfection (21). Clinicians encountering altered mental status should therefore prioritize the initiation of immediate ICU-level care, as neurological impairment is a critical prognostic indicator requiring urgent hemodynamic monitoring and organ support. Strikingly, *Candida albicans* was isolated solely from Patient 2, representing the only laboratory-confirmed fungal coinfection in this cohort. The incidence was 5.3% (1/19), which is lower than the 10% incidence reported in Japan; this may be attributed to the prudent use of corticosteroids and meticulous oral care protocols implemented for immunocompromised patients during active infection.

Although thrombocytopenia and elevated AST levels are characteristic features of SFTS, our study revealed no statistically significant associations between these parameters and clinical outcomes. This absence of prognostic correlation may result from the limited sample size and potential selection bias in case enrollment. Notably, while the majority of patients maintained their BUN levels within reference ranges, compared with survivors, nonsurvivors presented significantly elevated BUN levels. Additionally, prolonged APTT in patients who died was consistent with prior evidence establishing coagulopathy as a mortality predictor in SFTS progression. As the end product of protein metabolism, serial measurements of BUN not only reflect renal impairment but also correlate with emerging multiorgan dysfunction, particularly in patients with cardiovascular comorbidities where elevated BUN indicates increased mortality risk (22, 23). A longitudinal assessment of prognostic biomarkers in SFTS demonstrated that the predictive capacity of BUN exhibited progressive enhancement with disease progression, emphasizing the critical impact of renal impairment on late-stage outcomes. Furthermore, the study highlighted APTT as a persistent predictor throughout the clinical course, reflecting its crucial role in monitoring derangements of the intrinsic coagulation pathway (24).

Notably, our research revealed that an elevated viral load (>10^7^ copies/mL) is significantly correlated with fatal outcomes, which is consistent with previous studies identifying it as a mortality risk factor for DBV infections (25, 26). Mechanistically, increased viral titers markedly upregulate interferon-inducible protein 10 (IP-10) and macrophage inflammatory protein-1 (MIP-1) while hyperactivating immune cells to induce macrophage pyroptosis and initiate proinflammatory cytokine production, thereby triggering a cytokine storm that leads to multiorgan dysfunction and mortality (26, 27). Favipiravir, a GTP-competitive inhibitor of the viral RdRp, reduces CRF by inducing lethal mutagenesis that disrupts DBV replication, and this antiviral effect is more pronounced in patients with low baseline viral loads (28). Therefore, serial viral load monitoring not only facilitates severity assessment but also informs the optimal timing for antiviral treatment initiation.

The reported SFTS cases in Hubei Province are predominantly clustered in the northeastern region. The confirmed cases in our study were identified in Shiyan and Xiangyang cities, which are located in northwestern Hubei Province, enriching the regional distribution profile of DBV within the province. Previous studies have demonstrated transient viremic phases in caprine populations (29). In this study, one patient reported a history of ovine contact prior to symptom onset, suggesting that the precise transmission mechanisms governing DBV circulation between arthropod vectors and vertebrate reservoirs merit further investigation. Ding et al. conducted a case–control study across Henan, Hubei, and Shandong provinces in China and identified the presence of weeds and shrubs in occupational environments as risk factors for DBV infection (30). Furthermore, temperature and relative humidity may critically influence SFTS transmission dynamics in Hubei Province through their modulation of tick life cycles (31). Ticks, particularly *H. longicornis*, are established as the primary vectors of DBV. Adult *H. longicornis* can harbor DBV genetic material for up to 21 days, facilitating viral transmission (32). Among the 19 confirmed cases in this study, two patients reported definitive arthropod bite histories prior to symptom onset, while the majority had occupational exposure to farmlands/tea plantations or engaged in outdoor activities, thereby significantly increasing their risk of “tick exposure” (33).

Genotyping is a crucial tool for identifying differences in the immunogenicity, antigenicity, and pathogenicity of viruses (33). The segmented classification system (A–F) is currently the most widely adopted method for DBV genotyping. As the first reported and predominant endemic region of SFTS, China has significant genotypic diversity and geographic clustering of DBV strains, with all known genotypes found nationwide. Notably, Hubei Province displays the highest viral diversity, characterized by the cocirculation of multiple DBV genotypes (34). By 2023, nearly half of the DBV lineages submitted by China to the GenBank database were classified as lineage F (18). All three isolates in this study were identified as genotype F, aligning with the predominant genotype distribution in China, indicating persistent circulation of this genotype within tick populations (18). These findings improve our understanding of DBV epidemiology in Hubei Province and provide substantial genomic evidence for characterizing viral transmission dynamics in China.

Prior studies have suggested that the disparity in CFR across countries and regions might be correlated with circulating viral genotypes. For example, a South Korean study reported the highest CFR (43.8%, 21/48) among patients infected with genotype B-2, whereas genotype A exhibited the lowest CFR (10%, 1/10) (12). Furthermore, a study employing phylogenetic analysis of DBV genetic characteristics in Xinyang city, Henan Province, China, revealed that among the Chinese-derived clades (I-V), clade IV (genotype C3) exhibited the highest CFR (32.9%), and this mortality pattern may correlate with clade IV-specific mutations in the NS protein (S207P, Q245H) and Gc protein (V587I), which potentially amplify the production of inflammatory mediators, including CXCL10, G-CSF, and IL-6, thereby driving cytokine storm pathogenesis (35). Notably, clade IV demonstrated a distinct geographic distribution within Hubei Province. Our study revealed 19 SFTS patients with a CFR of 31.6% (6/19), exceeding the average CFR reported in China (5.3–16.2%) but remaining marginally lower than the 2023 SFTS CFR (46.2%) documented at our medical center (36).

Recombination serves as a key driver of DBV evolution and has the potential to generate novel genotypes with enhanced pathogenicity and transmissibility (37). While recombination events have been reported across all the genomic segments of DBV during its evolutionary trajectory, the phylogenetic analysis in this study revealed no detectable recombination signatures. We hypothesize that historical recombination may have occurred but conferred no competitive advantage to the viral progeny, thus failing to establish sustained transmission. These findings underscore that recombination events warrant ongoing surveillance through large-scale genomic monitoring to capture emergent variants with epidemiological significance.

In conclusions, we analyzed the clinical and epidemiological characteristics of 19 patients with confirmed SFTS in northwestern Hubei Province in 2024. Bleeding, consciousness disturbances, prolonged APTT, elevated BUN, and high viral load were identified as predictors of mortality. Three viral isolates, highly homologous to DBV reference sequences in GenBank, were classified into the genotype F clade. Overall, our study focused on the epidemiological characteristics and phylogenetic clusters of DBV in northwestern Hubei Province, thereby expanding the understanding of its epidemiological and genetic landscape across the province. Future research should prioritize comprehensive genomic characterization of local tick populations and implement longitudinal clinical surveillance of SFTS patients to elucidate the epidemiological landscape of DBV in this region.

## Data Availability

The datasets presented in this study can be found in online repositories. The names of the repository/repositories and accession number(s) can be found in the article.
